# Correlation of brain segmental volume changes with clinical parameters: a longitudinal study in multiple sclerosis patients

**DOI:** 10.1055/s-0043-1761492

**Published:** 2023-03-22

**Authors:** Neslihan Eskut, Ali Murat Koc, Asli Koskderelioglu, Ismail Dilek, Mustafa Agah Tekindal

**Affiliations:** 1 University of Health Sciences, Izmir Bozyaka Education and Research Hospital, Department of Neurology, Izmir, Turkey.; 2 Izmir Katip Celebi University, Ataturk Education and Research Hospital, Department of Radiology, Izmir, Turkey.; 3 University of Health Sciences, Izmir Bozyaka Education and Research Hospital, Department of Radiology, Izmir, Turkey.; 4 Izmir Katip Celebi University, Department of Biostatistics, Izmir, Turkey.

**Keywords:** Multiple Sclerosis, Magnetic Resonance Imaging, Social Network Analysis, Atrophy, Esclerose Múltipla, Imageamento por Ressonância Magnética, Análise de Rede Social, Atrofia

## Abstract

**Objective** To measure the cranial volume differences from 15 different parts in the follow-up of relapsing-remitting multiple sclerosis (RRMS) patients and correlate them with clinical parameters.

**Methods** Forty-seven patients with RRMS were included in the study. Patients were grouped into two categories; low Expanded Disability Status Scale (EDSS) (< 3; group 1), and moderate-high EDSS (≥ 3; group 2). Patients were evaluated with Beck Depression Inventory (BDI), Montreal Cognitive Assessment (MOCA), Symbol Digit Modalities Test (SDMT), Fatigue Severity Scale (FSS), and calculated Annualized Relapse Rate (ARR) scores. Magnetic resonance imaging (MRI) was performed with a 1.5T MRI device (Magnetom AERA, Siemens, Erlangen, Germany) twice in a 1-year period. Volumetric analysis was performed by a free, automated, online MRI brain volumetry software. The differences in volumetric values between the two MRI scans were calculated and correlated with the demographic and clinical parameters of the patients.

**Results** The number of attacks, disease duration, BDI, and FSS scores were higher in group 2; SDMT was higher in group 1. As expected, volumetric analyses have shown volume loss in total cerebral white matter in follow-up patients ( *p* < 0.001). In addition, putaminal volume loss was related to a higher number of attacks. Besides, a negative relation between FSS with total amygdala volumes, a link between atrophy of globus pallidus and ARR, and BDI scores was found with the aid of network analysis.

**Conclusions** Apart from a visual demonstration of volume loss, cranial MRI with volumetric analysis has a great potential for revealing covert links between segmental volume changes and clinical parameters.

## Resumo

**Objetivo** Medir as diferenças de dominância craniana de 15 regiões diferentes no seguimento de pacientes com esclerose múltipla recorrente-remitente (EMRR) e correlacioná-las com parâmetros clínicos.

**Métodos** Quarenta e sete pacientes com EMRR foram incluídos no estudo. Os pacientes foram agrupados em duas categorias; EDSS baixo (< 3; grupo 1) e EDSS médio-alto (≥ 3; grupo 2). Os pacientes foram avaliados com o Inventário de Depressão de Beck (BDI, na sigla em inglês), Montreal Cognitive Assessment (MOCA, na sigla em inglês), Symbol Digit Modality Tests (SDMT, na sigla em inglês), Fatigue Severity Scale (FSS, na sigla em inglês) e taxa de ataque anual (ARR, na sigla em inglês). Duas ressonâncias magnéticas (RMs) foram feitas em um ano com um aparelho de imagem de 1,5 T MR (Magnetom AERA, Siemens, Erlangen, Alemanha). A análise de volume foi realizada com um software de medição mestre cerebral de RM gratuito e automatizado. As diferenças volumétricas entre os dois exames de RM foram calculadas e correlacionadas com os parâmetros demográficos e clínicos dos pacientes.

**Resultados** Número de crises, duração da doença, escores BDI e FSS foram mais elevados no grupo 2; as pontuações do SDMT foram maiores no grupo 1. Como esperado, as análises volumétricas mostraram perda total de volume de substância branca no seguimento ( *p* < 0,001). Além disso, a perda da dominância putaminal foi associada ao maior número de ataques. Além disso, uma relação negativa entre FSS e volume total da amígdala, e uma correlação entre ARR e BDI e atrofia do globo pálido foi determinada com a ajuda da análise de rede.

**Conclusões** Além da demonstração visual da perda de volume, a RM com análise volumétrica tem grande potencial para revelar alterações segmentares dominantes e conexões ocultas entre parâmetros clínicos.

## INTRODUCTION

Multiple sclerosis (MS) is a chronic central nervous system disease characterized by demyelination, inflammation, and axonal degeneration. The most common MS subtype is relapsing-remitting MS (RRMS). It shows a progressive course over time with relapse and recovery periods. The evidence of axonal loss and neurodegeneration exists in the early course of the disease and causes permanent brain volume loss. In recent years, the relationship between brain atrophy and clinical parameters in MS patients has been the subject of many research areas. 1

With the help of new techniques, it is now possible to precisely measure the volume of the whole brain with its compartments in mm ^3^ with 3-dimensional (3D) magnetic resonance imaging (MRI) algorithms. 2 The present study aimed to evaluate the changes in brain volume and their relation with clinical parameters in patients with RRMS who remain clinically stable over 1 year.

## METHODS

### Study design and participants

The present prospective study included patients diagnosed with RRMS in the Multiple Sclerosis Unit of the University of Health Sciences, Izmir Bozyaka Education and Research Hospital between March 1, 2020 and September 9, 2021 clinically stable in their follow-up. We included patients aged between 18 and 65 years old who were followed up for at least 1 year. The patients who participated in the study had Expanded Disability Status Scale (EDSS) scores between 0 and 6.5. We excluded RRMS patients with any relapse in the last 3 months; illiterate patients presented relapse during the study period. Of the 95 patients, we excluded 25 with a relapse during the study period, 10 with a relapse in the last 3 months, 8 refused to have an MRI scan in the 1 ^st^ year, and 5 were illiterate. We assigned the study period to 18 months to include our the 1 ^st^ -year magnetic resonance imaging (MRI) of the participants of the study.

### Ethical approval

The institutional review board of the University of Health Sciences, Izmir Bozyaka Education and Research Hospital approved the present study (reference number: 1; date: November 27, 2020).

### Consent of the patients

All participants were included in the study after giving written informed consent.

### Clinical evaluation

All patients were evaluated with Beck Depression Inventory (BDI), Montreal Cognitive Assessment (MoCA), Symbol Digit Modalities Test (SDMT), Fatigue Severity Scale (FSS), the Expanded Disability Status Scale (EDSS) was calculated, and Annualized Relapse Rate (ARR) scores at the time of admission. Patients' age, gender, body mass index (BMI), smoking status, and education levels were recorded. We grouped patients into 2 groups according to EDSS scores; group 1, low level of disability (EDSS < 3), and group 2 moderate or high level of disability (EDSS ≥ 3).

### Study instruments

#### Expanded Disability Status Scale (EDSS)

We used the EDSS to assess disability in patients with MS. It is widely used in clinical practice and clinical trials, as Kurtzke et al. reported. 3

#### Beck Depression Inventory (BDI)

Depressive symptoms were evaluated with the BDI. The BDI is recommended as a screening test to identify depression in patients with MS. Questions addressed depressive symptoms such as failure, dissatisfaction, pessimism, feelings of guilt, restlessness, poor appetite indecision, fatigue, sleep disorders, and social withdrawal – the total score ranged from 0 to 63. A score between 30 and 63 indicates severe depression; 18 to 29 moderate depression; and 11 to 17 mild depression. 4

#### Montreal Cognitive Assessment Test (MoCA)

The MoCA is a psychometrically valid screening tool in patients with MS to assess MS-related cognitive impairment. Using the MoCA allows a global cognitive measurement including attention, concentration, short-term memory, executive functions, delayed recalling, language, visuospatial abilities, conceptual thinking, calculations, and orientation. High MoCA scores represent good cognitive status; scores > 26 were accepted as normal. 5

#### Symbol Digit Modality Test (SDMT)

The SDMT is important and sensitive to detect MS-associated cognitive impairment. The SDMT is performed to measure information attention, concentration, and processing speed. Higher scores specify better cognitive processing speed. 6

#### Fatigue Severity Scale (FSS)

The FSS, a validated tool in patients with MS, was first published in 1989 by Krupp et al.. 7 We assessed the existence and severity of fatigue using the FSS. The FSS is a self-report questionnaire that consists of 9 items with 7 levels of response from 1 to 7. Fatigue severity scales scores < 2.8 were considered normal, and > 6.1 were considered chronic fatigue syndrome.

#### Imaging and volumetric analysis

**Figure 1.**
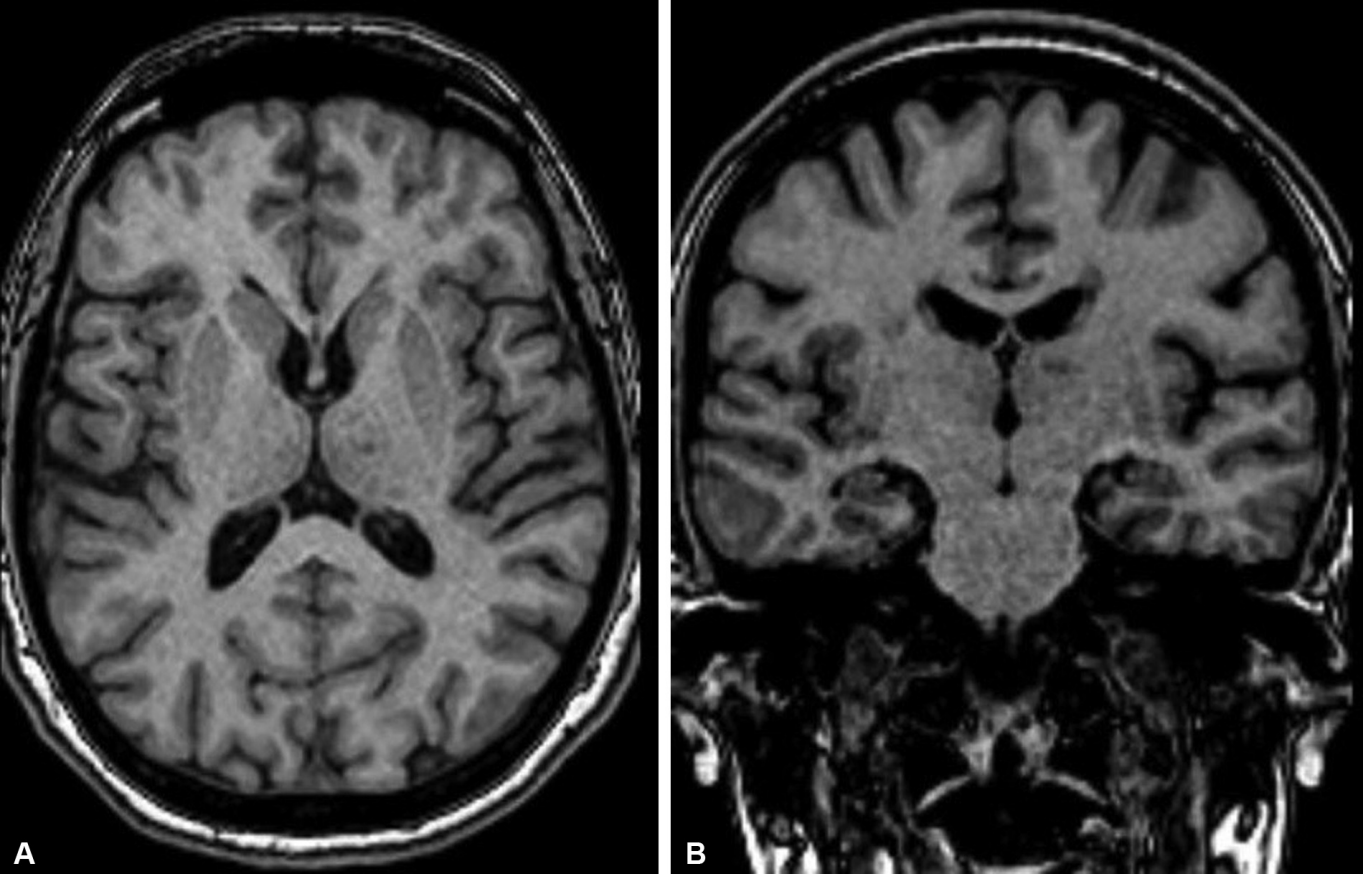
A. High resolution 3D-T1 MR images in axial plane; B. High resolution 3D-T1 MR images in coronal planes.

Magnetic resonance imaging was performed with a 1.5T MRI device (Magnetom AERA, Siemen, Erlangen, Germany) twice for each patient with the MS dedicated protocol at a 1-year interval. Precontrast T1W images were obtained with a 1-mm slice thickness covering the whole brain ( Figure 1 A and B ).

**Figure 2.**
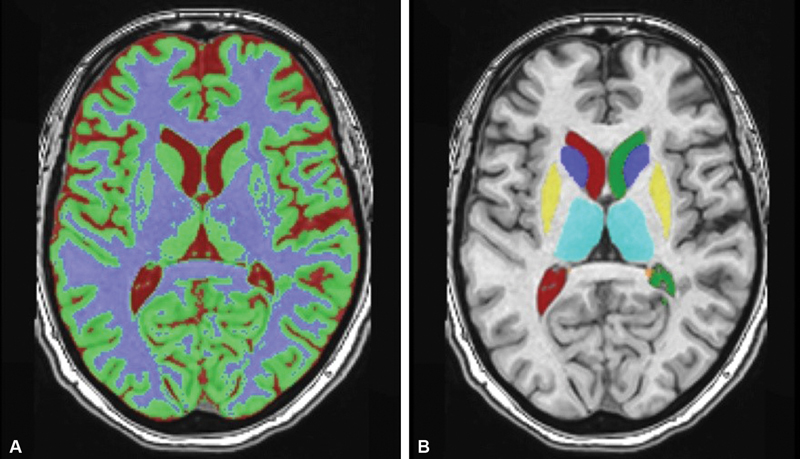
A. An example of tissue classification of subcortical segments; B. An example of tissue labelling of subcortical segments.

After anonymization and conversion to NIfTI format, volumetric analysis was performed by a free automated online MRI brain volumetry software (volBrain. https://volbrain.upv.es/ ). 8 9 Cerebral and cerebellar gray matter, white matter, total intracranial volume, volumes, and percentages of the brainstem, thalamus, globus pallidus, caudate nucleus, putamen, amygdala, nucleus accumbens, hippocampus, and ventricles were calculated ( Figure 2 A and B ).

The differences in these compartments between the two MRI scans were calculated. A cutoff value of 0.5% was used to interpret the volume changes and categorize the Volbrain outcomes. 10 Differences smaller than these values were included in the stable category. Atrophy of intracranial compartments was investigated at the baseline volumetric MRI according to the population averages specified by age and gender. The differences in volumetric values between the two MRI scans were calculated and correlated with the demographic information and clinical parameters of the patients. Descriptive statistics were also analyzed in two categories of EDSS scores.

### Statistical analysis

Data are presented as several observations ( *n* , %), mean ± standard deviation (SD), range. We used the results of homogeneity (Levene test) and normality (Shapiro-Wilk test) to decide the statistical methods for comparing the study groups. Among normally distributed groups with homogeneous variances, the student t-test was used to compare independent groups. According to the test results, when parametric test assumptions were unavailable for some variables, the independent groups were compared using the Mann-Whitney-U test. Among normally distributed groups, dependent groups were compared using the paired t-test. According to the test results, for some variables, parametric test assumptions were not available; therefore, the dependent groups were compared using the Wilcoxon test. We analyzed the categorical data using the Fischer exact test and chi-squared tests. In cases where the expected counts for inclusion were not met in < 20% of the cells, we used the "Monte Carlo Simulation Method" and the values were determined. The network construction, visualization, and analysis were performed using the open-source 'R' statistical software 11 and the R-packages' bootnet' and 'qgraph' in particular.

For the significance level of the tests, *p* < 0.05 and *p* < 0.01 values were accepted.

## RESULTS

Forty-seven patients diagnosed with RRMS and without cognitive complaints were included in the study. The mean age of the participants was 39.53 ± 9.07 years old, and 51.1% ( *n* = 24) of them were male. The demographic information and clinical evaluation scores of the participants are summarized in Table 1 .

**Table 1:** Demographic and clinical information of the MS patients

	Mean ± SD	Median (Min-Max)
Age (years)	39.53 ± 9.07	39(23–63)
BMI (kg/m ^2^ )	25.18 ± 4.32	25.4(16.70–34.10)
Education (years)	9.72 ± 3.57	11(5–16)
EDSS (0–10)	2.37 ± 1.69	2(0–6.5)
Number of attacks	4.51 ± 3.29	4(1–18)
Disease duration (years)	9.07 ± 6.23	8(1–22)
ARR	0.57 ± 0.30	0.5(0.2–1.30)
BDI (0–63)	14.87 ± 11.44	12(0–41)
FSS (1–7)	4.20 ± 2.20	4.6(0–7)
MOCA (0–30)	25.0 ± 2.13	25(17–30)
SDMT	36.20 ± 12.83	36(12–62)

Abbreviations: ARR, annualized relapse rate; BDI, Beck Depression Inventory; BMI, Body Mass Index; EDSS, Expanded Disability Status Scale; FSS, Fatigue Severity Scale; MOCA, Montreal Cognitive Assessment; MS, Multiple Sclerosis; SD, Standard Deviation; SDMT, Symbol Digit Modalities Test.

The EDSS score of 57.4% of the patients was < 3 points ( *n* = 27). When the patients are grouped into two according to the EDSS scores, the number of attacks, duration of the disease, BDI, and FSS scores of those with an EDSS score ≥ 3 are significantly higher than those of patients with an EDSS score < 3 (p-value < 0.001; 0.003; 0.005; 0.011 respectively). In addition, the SDMT value was significantly higher in patients with an EDSS score < 3 ( *p =* 0.021) ( Table 2 ).

**Table 2:** Distribution of clinical data in two groups of MS patients according to the EDSS scores

	EDSS		*p-value*
	Group 1 (< 3) Mean ± SD (Median [Min-Max])	Group 2 (≥ 3) Mean ± SD (Median [Min-Max])	
Age (years)	39.15 ± 9.86 (39 [23–63])	40.05 ± 8.10 (40 [26–52])	0.613
BMI (kg/m ^2^ )	25.05 ± 4.3 (25.6 [16.7–33.8])	25.36 ± 4.46 (24.5 [17.8–34.1])	0.821
Education (years)	10.59 ± 3.55 (11 [5–16])	8.55 ± 3.32 (8 [5–15])	0.062
Number of attacks	3 ± 2.02 [2(1–9])	6.55 ± 3.60 (6 [1–18])	**< 0.001**
Disease duration (years)	6.76 ± 5 (5 [1–20])	12.20 ± 6.48(11.5 [1–22])	**0.003**
ARR	0.54 ± 0.30 (0.5 [0.2–1.3])	0.62 ± 0.30 (0.5 [0.3–1.3])	0.295
BDI (0–63)	11.07 ± 10.16 (9 [0–41])	20 ± 11.27 (20 [1–38])	**0.005**
FSS (1–7)	3.49 ± 2.13 (3.4 [0–7])	5.17 ± 1.96 (5.7 [1–7])	**0.011**
MOCA (0–30)	25.26 ± 1.46 (25 [22–30])	24.65 ± 2.81(25 [17–28])	0.866
SDMT	40.11 ± 13.26 (41 [12–62])	30.9 ± 10.34 (33 [13–69])	0.021

Abbreviations: ARR, annualized relapse rate; BDI, Beck Depression Inventory; BMI, Body Mass Index; EDSS, Expanded Disability Status Scale; FSS, Fatigue Severity Scale; MOCA, Montreal Cognitive Assessment; MS, Multiple Sclerosis; SD, Standard Deviation; SDMT, Symbol Digit Modalities Test.

Note: Significant *p* values are presented in bold ( *p* < 0.05; *p* < 0.01).

The neuroradiological imaging analysis has evaluated the significant changes in the brain volumetry between two MRI scans. In the follow-up, we found a significant decrease in the volume of cerebral white matter (p <0.001). Furthermore, there was a significant decrease in the total putaminal volume ( *p* = 0.041). Table 3 shows the detailed volumetric data. including comparing two MRI scans.

**Table 3:** Comparison of volumetric analysis results of intracranial compartments between two MRI scans

	1 ^st^ MRI		2 ^nd^ MRI		*p-Value*
Volumetric analysis of intracranial compartments(cm ^3^ )	Mean ± SD	Median (Min-Max)	Mean ± SD	Median (Min-Max)	
Tissue WM	455.81 ± 120.21	452.38 (112.48–911.40)	455.93 ± 84.91	461.77(251.80–682.29)	0.478 ^*^
Tissue GM	670.10 ± 114.36	687.25(236.45–829.60)	671.03 ± 69.81	683.92(524.01–844.34)	0.363 ^*^
Tissue Brain	1147.45 ± 136.92	1139.59(841.64–1448.44)	1136.95 ± 129.04	1133.88(841.14–1460.67)	0.245 ^**^
Cerebrum WM	569.14 ± 98.32	578.11(197.70–712.09)	409.96 ± 75.44	413.33(235.98–599.73)	< 0.001 ^*^
Cerebrum GM	430.45 ± 131.04	410.60(240.37–1017.21)	577.44 ± 63.01	573.17(433.39–738.17)	< 0.001 ^*^
Cerebrum total	999.60 ± 125.91	982.04(718.88–1264.29)	987.40 ± 116.579	980.04(716.04–1299.25)	0.427 ^*^
Brain Stem	22.0 ± 2.79	21.95(15.83–29.11)	22.92 ± 2.79	21.73(16.0–29.22)	0.280 ^*^
Caudate Nucleus	6.19 ± 0.88	6.24(4.22–8.02)	6.12 ± 0.87	6.08(4.32–8.47)	0.228 ^**^
Putamen	7.53 ± 0.96	7.37(5.73–10.05)	7.41 ± 0.93	7.41(5.58–10.45)	0.041 ^**^
Thalamus	9.01 ± 1.65	9.17(4.05–12.64)	9.01 ± 1.64	9.18(3.98–12.49)	0.975 ^**^
Glomus Pallidus	1.97 ± 0.41	2.02(0.99–2.78)	1.95 ± 0.37	1.98(1.15–2.65)	0.505 ^*^
Hipocampus	7.63 ± 0.90	7.59(5.49–9.59)	7.62 ± 0.86	7.63(5.50–9.53)	0.895 ^*^
Amygdala	1.33 ± 0.26	1.38(0.81–1.97)	1.35 ± 0.22	1.36(0.86–1.79)	0.210 ^*^
Nuc. Accumbens	0.55 ± 0.94	0.543(0.339–0.777)	0.54 ± 0.79	0.54(0.38–0.77)	0.242 ^**^
Cerebellum	125.84 ± 16.80	126.43(86–173.72)	127.68 ± 14.94	128.27(97.73–175.58)	0.176 ^**^

Abbreviations: GM, grey matter; SD, Standard Deviation; WM, white matter.

Notes: Values are given in cm3. Significant p values are presented in bold ( *p* <0.05; *p* <0.01); *Wilcoxon test; **Paired t-test.

Further analysis has evaluated the possible relations with volume changes in the brain. We found no difference in age, BMI, education, number of attacks, disease duration, and the ARR in patients with decreased total white matter volume among patients without a decrease in the follow-up period. Besides, patients with a significant decrease in total white matter volume had similar BDI, FSS, MoCA, and SDMT scores compared with patients without a decrease ( *p* > 0.05 for all). On the other hand, decreased putaminal volume was significantly related to more attacks. The median number of attacks was 5 (1 to 18) for those whose putaminal volumes were decreased in the follow-up and 2.5 (1 to 10) for those whose putaminal volumes did not decrease (p = 0.036).

**Figure 3.**
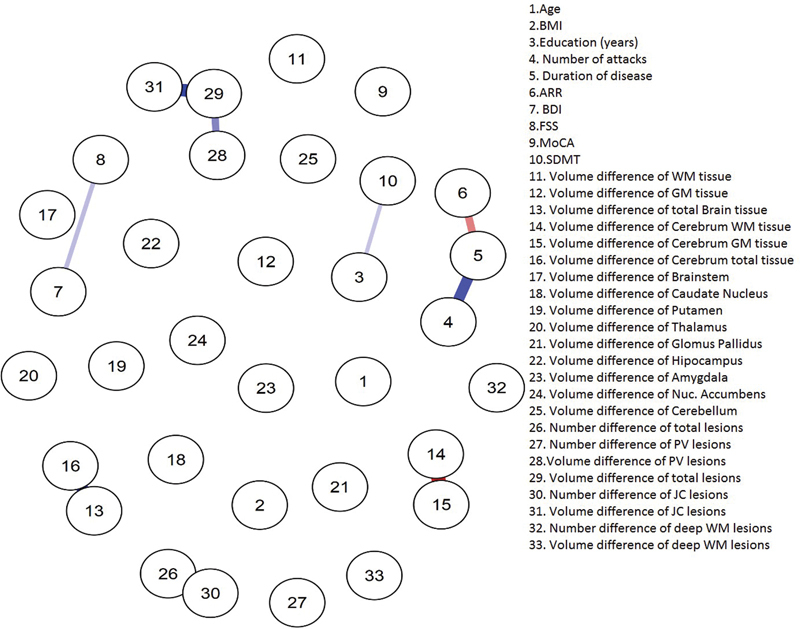
Network analyzed data of EDSS (<3) variables. Abbreviations: ARR, Annualized relapse rate; BDI, Beck Depression Inventory; BMI, Body mass index; FSS, Fatigue Severity Scale; GM, Grey matter; JC, Juxtacortical; MOCA, Montreal Cognitive Assessment; SDMT, Symbol Digit Modalities Test; PV, Periventricular; WM, White matter. Note: Values are given in cm3.

**Figure 4.**
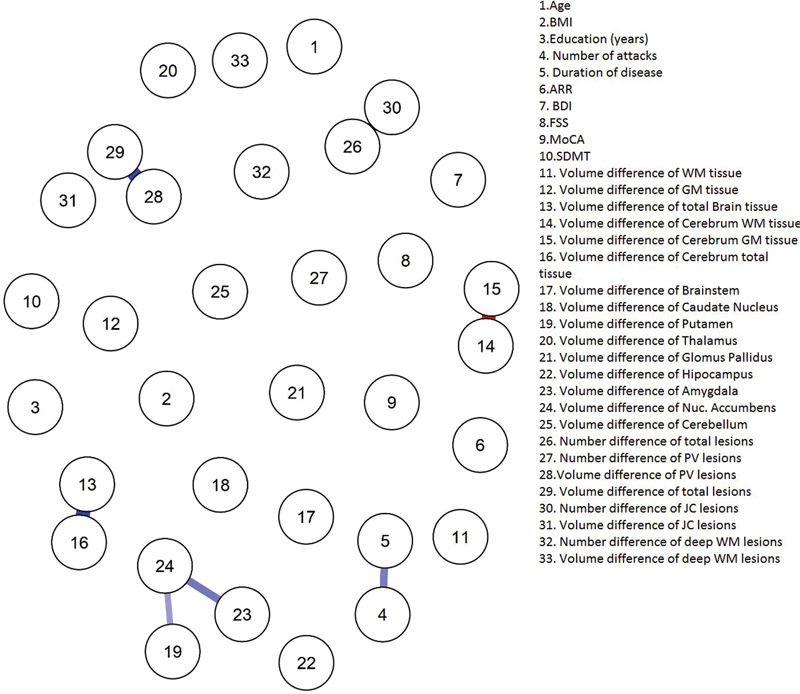
Network analyzed data of EDSS (≥3) variables. Abbreviations: ARR, Annualized relapse rate; BDI, Beck Depression Inventory; BMI, Body mass index; FSS, Fatigue Severity Scale; GM, Grey matter; JC, Juxtacortical; MOCA, Montreal Cognitive Assessment; SDMT, Symbol Digit Modalities Test; PV, Periventricular; WM, White matter. Values are given in cm3.

Network analysis has shown several statistically significant links between volumes of different compartments in initial MRI and clinical scores. There were negative correlations between the number of attacks and total volumes of white matter, cerebral tissue, caudate nucleus, and thalamus. Likewise, negative correlations were found between disease duration and brainstem, caudate nucleus, putamen, thalamus, and globus pallidus volumes. The FSS score was negatively related to total amygdala volume ( Figure 3 and 4 ).

Gaussian graphical models have shown that disease duration was negatively connected to ARR while positively connected to the number of attacks in group 1. Also, BDI and FSS scores were positively connected. In group 2, the volume changes of total cerebral and total intracranial tissue (gray and white matter) were positively connected. Also, the volume changes in the nucleus accumbens were positively connected with volume changes of the total amygdala and putamen. Similarly, disease duration was positively connected to the number of attacks.

Analysis of the plaque number showed an increase in mean values of total plaque number and volume, but no statistically significant difference was found ( *p* = 0.058 and *p* = 0.512). Subgroup analysis also showed no statistically significant change in the number and volumes of plaques in different compartments; periventricular, juxtacortical, and deep white matter.

## DISCUSSION

In the present prospective study, a longitudinal 3D volumetric MRI analysis, several clinical tests and examinations were performed for the RRMS patients. Our results have shown expected volumetric changes, that is, white matter atrophy. On the other hand, we have outlined essential connections between volumetric changes and clinical scores that might broaden the anatomical changes in the brains of MS patients.

Kurtzke's EDSS is the most commonly used clinical scoring system in the follow-up of MS patients. 3 12 It is often used as a standard prognosis parameter and correlated with other tests and radiological results. A threshold of 3 was used in our study to differentiate patients into groups of low disability (Group 1; *n* = 27) and moderate-to-high levels of disability (Group 2; *n* = 20). Since the progressive nature of demyelination, the longer the disease duration and the higher number of the attacks results in continuous damage in the brains of MS patients. As expected, we have found higher EDSS scores in patients with a higher number of attacks and longer disease duration. The BDI and FSS scores also have well-known correlations with the same parameters. Following these findings, cognitive test scores with SDMT were higher in Group 1. 13 14 15

Magnetic resonance imaging is the primary imaging method in MS patients that provides excellent information about the presence, characterization, and the temporal and spatial distribution of MS lesions. A sensitive volumetric analysis can be performed with 3D volumetric MR imaging, the standard imaging protocol in many centers. Atrophy of the whole brain and its components can be determined with 3D-MRI methods.

Multiple sclerosis has been shown to affect brain volumes distinct from healthy aging individuals. Earlier beliefs about MS affecting white matter have also been resolved in time. Volume changes in white and gray matter, specifically deep gray matter areas, have been observed in many studies. 1 10 16 17 18 Longitudinal MRI studies have become more important in identifying the volume change in specific brain areas and correlating them with present and future clinical evaluation results. 19 The present study has observed a major white matter volume loss in one year in RRMS patients. However, contrary to the literature, we could not observe a total gray matter volume loss. 20 21 This finding might have occurred due to the volumetric analysis software parameters.

Furthermore, measuring the gray matter volume could be more appropriate based on samples from different areas and cortical thicknesses. 21 Fisher et al. explained the possible errors related to GM calculation in their longitudinal MRI study. 22 On the other hand, we have found a subcortical GM atrophy in both putamens, which is also significantly correlated with the number of attacks. As Eshaghi et al. have postulated, the putamen has known motor cortex inputs, retrograde demyelination of this area may appear prominent in RRMS patients due to the relatively high lesion load occurring from the attacks. 23 Putaminal atrophy also correlates with the clinical parameters and the progress of the disease. 24 In our study, the putaminal volume average of patients in Group 1 did not show atrophy, unlike the patients in Group 2 at the annual follow-up MRI scan.

The baseline clinical scores were not directly correlated with either white matter atrophy or the other volumetry results in our study. Instead, pattern and network analysis have revealed negative relations between FSS and total amygdala volume. This finding can be explained by the contribution of the amygdala to social cognition, which affects the FSS scores when impaired. 25 Following the findings of Batista et al., we have also outlined a positive relation between BDI and FSI scores. 26 Another interesting finding was the link between ARR and BDI in patients with globus pallidus atrophy. Supportively, Stuke et al. have found a similar association between atrophy of right globus pallidus and worsening of depressive symptoms over time. 27 An increasing number of attacks and atrophy of globus pallidus over time were associated with depressive symptoms in our study. The disrupted reward system can explain depressive symptoms such as anhedonia and apathy in MS patients with globus pallidus atrophy in the dopaminergic circuit. 28

There are several strengths of our study. We prospectively included RRMS patients who were clinically stable and evaluated the volumetric changes of intracranial structures in detail. Additionally, the overall study population and subgroups were relatively homogeneous according to the EDSS. Moreover, network analysis revealed possible links between segmental volume changes and clinical parameters in 1 year. However, there had been various study limitations that should be kept in mind. Although major confounding factors like the scan parameters and the MRI machine were all fixed, factors like scanning time of the day, scan-rescan variability, and gradient distortion might have affected our results. Besides, we aimed to analyze volumetric changes; we could not evaluate the plaque count or lesion load due to software issues. We conducted MRI parameters of two scans in 1 year. Due to the short follow-up period, obvious volume changes might not have emerged. In addition, we included a limited number of RRMS patients without any changes in the clinical course.

In conclusion, our prospective study demonstrated a significant decrease in cerebral white matter and total putamen volumes in the 1-year MS patients who were clinically stable. In addition, the network analysis contributes well to the understanding of possible links between segmental volume changes and clinical parameters. Volumetric cranial MRI allows both diagnostic semi-quantitative visual analysis and automatic quantitative measurement during the follow-up of MS patients.
